# H_2_S promotes flowering in *Brassica rapa* ssp. *pekinensis* by persulfidation of the splicing factor BraATO2

**DOI:** 10.1093/hr/uhaf190

**Published:** 2025-07-16

**Authors:** Xuefeng Hao, Weier Li, Haiyan Cao, Fulin Tang, Tian Ma, Jiao Zhang, Liping Zhang, Limei Chen, Zhuping Jin, Yanxi Pei

**Affiliations:** College of Biological Sciences and Technology, Taiyuan Normal University, 319 Daxue Street, Yuci District, Jinzhong, Shanxi Province 030619, China; School of Life Science, Shanxi Key Laboratory for Research and Development of Regional Plants, Shanxi University, Nanzhonghuan East Street No. 63, Xiaodian District, Taiyuan, Shanxi Province 030032, China; College of Biological Sciences and Technology, Taiyuan Normal University, 319 Daxue Street, Yuci District, Jinzhong, Shanxi Province 030619, China; School of Life Science, Shanxi Key Laboratory for Research and Development of Regional Plants, Shanxi University, Nanzhonghuan East Street No. 63, Xiaodian District, Taiyuan, Shanxi Province 030032, China; School of Life Science, Shanxi Key Laboratory for Research and Development of Regional Plants, Shanxi University, Nanzhonghuan East Street No. 63, Xiaodian District, Taiyuan, Shanxi Province 030032, China; School of Life Science, Shanxi Key Laboratory for Research and Development of Regional Plants, Shanxi University, Nanzhonghuan East Street No. 63, Xiaodian District, Taiyuan, Shanxi Province 030032, China; School of Life Science, Shanxi Key Laboratory for Research and Development of Regional Plants, Shanxi University, Nanzhonghuan East Street No. 63, Xiaodian District, Taiyuan, Shanxi Province 030032, China; State Key Laboratory of Plant Environmental Resilience, College of Biological Sciences, China Agricultural University, Yuanmingyuan West Road No. 2, Haidian District, Beijing 100193, China; School of Life Science, Shanxi Key Laboratory for Research and Development of Regional Plants, Shanxi University, Nanzhonghuan East Street No. 63, Xiaodian District, Taiyuan, Shanxi Province 030032, China; College of Biological Sciences and Technology, Taiyuan Normal University, 319 Daxue Street, Yuci District, Jinzhong, Shanxi Province 030619, China; School of Life Science, Shanxi Key Laboratory for Research and Development of Regional Plants, Shanxi University, Nanzhonghuan East Street No. 63, Xiaodian District, Taiyuan, Shanxi Province 030032, China

## Abstract

Hydrogen sulfide (H_2_S) is a newly identified gasotransmitter that plays an irreplaceable physiological role in plant growth, development, and environmental responses through persulfidation of cysteine (Cys) residues (RSSH). However, reports on the direct RSSH targets of H_2_S in plants remain limited. The flowering regulation mechanisms of *Brassica rapa* ssp. *pekinensis* are a significant scientific issue in the crop production industry; however, they remain poorly understood. BraATO2 is an important splicing factor in genetic alternative splicing (AS). Our study demonstrated that H_2_S regulated BraATO2 function by persulfidating the Cys residue at position 416. In turn, this influenced the AS patterns of multiple genes in *B. rapa*, specifically the flowering regulator *BraAGL31*/*MAF2* within the *FLOWERING LOCUS C*-like (*FLC*-like) gene family, causing accelerated flowering. This study identified a new direct target of H_2_S and uncovered a novel pathway influencing flowering in *B. rapa.* Furthermore, the study findings provide fresh insights into the development of innovative flowering regulators for plants.

## Introduction

*Brassica rapa* ssp. *pekinensis* originated in China and is one of the most important vegetable crops in East Asia [1, 2]. The completion of the *B. rapa* Genome Sequencing Project in 2011 and the establishment of the *Brassica* database (http://brassicadb.cn) have provided strong knowledge base for molecular biology research of *B. rapa* [3]. A significant collinearity relationship exists between the *B. rapa* and *Arabidopsis thaliana* genomes, which facilitates the utilization of the rich functional genetic information on *Arabidopsis* for molecular physiology research on *B. rapa* [3–5]. The flowering regulation mechanism in *B. rapa* is a significant scientific matter in the crop production industry [6], given the varying of breeders and growers regarding the timing of flowering. However, the current understanding of the flowering regulation mechanisms in *B. rapa* remains quite limited.

Hydrogen sulfide (H_2_S) is a colorless, toxic gas with a distinctive odor akin to rotten eggs. Its fundamental characteristics and physiological functions as a gaseous signaling molecule were gradually elucidated around the 2000s. H_2_S is considered the third most significant gasotransmitter after nitric oxide (NO) and carbon monoxide (CO) [7, 8]. It is crucial in plant growth and development, particularly in the stress response [9], and its effects are dose dependent [10]. At physiological concentrations, H_2_S promotes seed germination, influences root morphology establishment, enhances leaf photosynthesis, and delays senescence. Furthermore, it facilitates plant tolerance to abiotic stresses, including drought, salinity, and heavy metal toxicity [9, 11, 12]. The progress of research on H_2_S has gradually revealed its diverse physiological roles. Accordingly, the underlying regulatory mechanisms of H_2_S on plant growth and development have garnered significant scientific interest. Among such mechanisms under research is the ability of H_2_S to induce persulfidation modifications of cysteine (Cys) residues in proteins (RSSH), altering their conformation and thus regulating the activity, structure, and subcellular localization of target proteins [13–15]. This posttranslational covalent modification of functional proteins by H_2_S has been increasingly confirmed in various studies [16] and bears a striking resemblance to the action mechanism of NO. The earliest report describing such mechanism was in 2009, which first confirmed that RSSH modification enhances the activity of the glyceraldehyde 3-phosphate dehydrogenase (GAPDH) enzyme, thereby promoting actin polymerization [17]. Aroca *et al*. [18] demonstrated that H_2_S modulates the binding activity of phospholipids through the RSSH modification of ATG18a, causing changes in autophagosome abundance and size. In tomatoes, H_2_S can RSSH modify the 1-aminocyclopropane-1-carboxylic-acid oxidase (ACO) enzymes LeACO1 and LeACO2, thereby reducing enzyme activity and regulating ethylene biosynthesis [19]. Studies on *B. rapa* found that H_2_S can RSSH modify the large subunit of ribulose 1,5-bisphosphate carboxylase/oxygenase (BrRuBisCO), enhancing the photosynthetic efficiency of *B. rapa* leaves to resist drought stress [8]. In *Arabidopsis,* abscisic acid (ABA)-induced RSSH modifications of desulfhydrase (DES) promote H_2_S production and facilitate stomatal closure [15, 20]. Another study found that H_2_S can mediate the RSSH modification of Cys250 in the positive regulatory factor abscisic acid insensitive 4 (ABI4) within the ABA signaling pathway, which enhances the transcriptional activation of the downstream target mitogen-activated protein kinase kinase kinase 18 (MAPKKK18). H_2_S signaling regulates the ABA response in plants through this pathway [21]. Furthermore, H_2_S-mediated RSSH modification can alter the conformation of the key kinase protein sucrose nonfermenting 1-related protein kinases 2.6 (SnRK2.6), which are activated by the ABA-induced inhibition of type-2C protein-phosphatases in the ABA signaling pathway, thereby increasing the efficiency of transferring ATP-γ-phosphate groups and enhancing kinase activity [22–24]. RSSH modification can also affect root hair development in plants by disrupting normal actin polymerization [25].

Alternative splicing (AS), also called variable splicing, refers to the process by which precursor mRNA undergoes diverse splicing patterns to generate various mRNA isoforms [26, 27], enabling a single gene to produce multiple products. AS is a crucial step in the posttranscriptional regulation of gene expression that contributes to the diversity in transcripts and increases the coding potential of the genome. The spliceosome is a large ribonucleoprotein complex that recognizes splice sites and branch points from which to splice pre-mRNA. During plant growth and development, organisms encounter complex external environments, which stimulate continuous changes in the structure, function, and subcellular localization of functional proteins. Splicing factors (SFs) are essential participants for assembling splicing precursors during this process [27, 28].

Our previous research has demonstrated that the application of H_2_S significantly promotes flowering in *B. rapa* and *A. thaliana*. Transcriptomic analysis revealed alterations in the AS patterns of numerous flowering-related genes in *B. rapa* treated with H_2_S [29]. Furthermore, our latest findings indicate that H_2_S can modify U2AF65a in *Arabidopsis* through RSSH, thereby altering the AS patterns of downstream genes and regulating flowering time [30]. In 2017, Aroca *et al*. [18] elucidated the regulatory effects of H_2_S on protein functions in various biological processes of *Arabidopsis* through the proteomic analysis of persulfidated proteins. Therefore, we speculate that influencing the AS pattern of certain genes through RSSH modification of certain AS factors is an important pathway by which H_2_S exerts its physiological functions. However, a comprehensive and systematic report on the SFs influenced by H_2_S has not yet been conducted.

Notably, the impact of H_2_S on flowering is only partially dependent on U2AF65a, and not all AS genes are associated with U2AF65a. Based on the results of transcriptomic data analysis, we speculate the existence of other splicing proteins that act as direct targets of H_2_S that influence flowering in *B. rapa*. This intriguing scientific question has captured our attention. To make breakthroughs in this area, the RSSH modification target SF proteins of H_2_S need to be identified. Given the complexity of the *B. rapa* genome, we first focused on the model plant *Arabidopsis*. Through methods, such as predicting H_2_S polysulfuration substrates, analyzing AS patterns in the transcriptome, and predicting molecular interactions, and reviewing related literature, we identified seven SF proteins in *Arabidopsis* as potential targets for H_2_S RSSH modifications: AtCC1 (AT2G16940), AtCC1-like (AT5G09880), AtATO (AT5G06160), AtRSZ22A (AT2G24590), AtMOS4 (AT3G18165), AtCBP80 (AT2G13540), and AtSWAP (AT1G14650). Because of the dynamic nature of spliceosomes and the restrictions of plant AS research systems, most studies have mainly investigated the structures of proteins and implemented omics approaches. However, the specific biological functions and mechanisms of these proteins remain largely unknown. This study conducted *in vitro* RSSH modification testing on the seven specific SFs using the collinear genome relationship between *A. thaliana* and *B. rapa*. Subsequently, we analyzed the RSSH modifications in target proteins and evaluated the splicing patterns of downstream genes to identify a novel direct target mediating the effect of H_2_S on flowering time in *B. rapa*. Our findings will facilitate the identification of new pathways of H_2_S activity and offer profound insights into the regulatory mechanisms governing flowering time in *B. rapa*.

## Results

### Application of exogenous H_2_S promoted flowering in *B. rapa*

**Figure 1:**
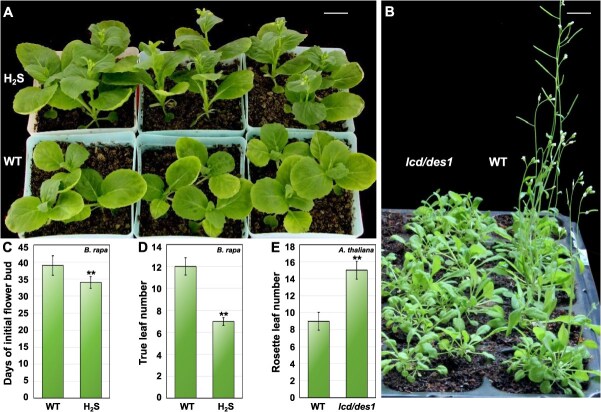
H_2_S promoted flowering in *Brassica rapa* and *Arabidopsis thaliana*. (A) Application of H_2_S (NaHS as donor) accelerated flowering in *B. rapa* (scale bars = 2.5 cm). (B) Comparison of flowering times between the *lcd/des1* double mutant (deficient in endogenous H_2_S-producing enzymes L-Cys desulfhydrase and desulfhydrase 1) and wild-type (WT) *Arabidopsis* plants (scale bars = 1.5 cm). Statistical analysis of C initial flower bud days (C), true leaf count in *B. rapa* (D), and rosette leaf count at flowering in both WT and *lcd/des1 A. thaliana* (E). WT: 0 μM H_2_S treatment; H_2_S: 100 μM H_2_S treatment. Data are expressed as the mean ± standard error (SE) of three independent experimental replicates (^**^P < 0.01, Student’s *t* test; 6–10 for each *B. rapa* replicate; 10–15 for each *A. thaliana* replicate).

Our preliminary research [30] indicated that H_2_S promoted flowering in *Arabidopsis.* The *lcd/des1* mutant, which lacks the endogenous enzymes l-Cys desulfhydrase and desulfhydrase 1 (DES1) necessary for H_2_S production [31], exhibited a notable late flowering phenotype (Fig. 1B), with the flowering time delayed from 9 to 15 rosette leaves (Fig. 1E). Consistent with these findings, the exogenous application of 100 μM H_2_S significantly accelerated flowering in *B. rapa* (Fig. 1A). Specifically, the initial flower bud emerged earlier in the treated group, from 39 to 34 days (Fig. 1C). Furthermore, the number of true leaves decreased from 12 to 7 (Fig. 1D). The differences in the timing of flower bud emergence and number of true leaves between the treated and control groups were significant.

### Analysis of RSSH modifications in splicing proteins as potential H_2_S targets in *Arabidopsis*

Previous transcriptome data from our laboratory indicate that H_2_S affects the AS patterns of numerous genes [30]. The literature shows that in the *Arabidopsis* proteome, approximately 5% of proteins may undergo RSSH modifications. These proteins participate in a wide range of biological functions, regulating critical processes such as carbon metabolism, RNA translation, and the plant response to abiotic and biotic stresses; however, conclusive experimental evidence is lacking [18]. Therefore, we speculate that H_2_S may regulate flowering in plants by influencing the AS patterns of genes through the RSSH modification of certain pre-mRNA splicing-related proteins. Because the literature on *B. rapa* is limited, we performed preliminary tests and screening using *A. thaliana*.

**Figure 2:**
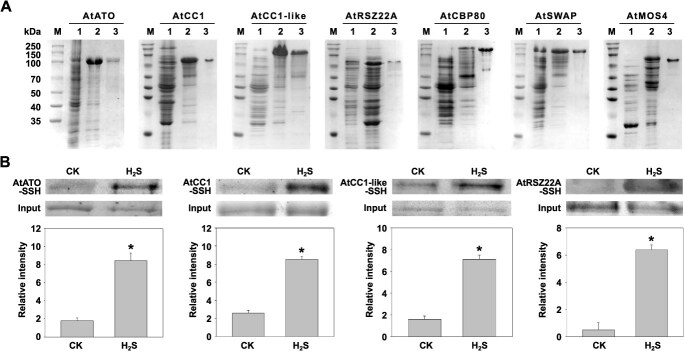
Detection of RSSH modifications in candidate splice factor proteins in *Arabidopsis*. (A) Induction and purification of recombinant proteins: pCold-*AtATO*, XF245-*AtCC1,* XF245-*AtCC1-*like*,* pCold-*AtRSZ22A*, pCold-*AtCBP80*, pCold-*AtSWAP*, and pCold-*AtMOS4* (M, protein molecular weight marker; 1, proteins from uninduced *Escherichia coli*; 2, proteins from isopropyl β-D-thiogalactoside (IPTG)-induced *E. coli*; 3, purified proteins). (B) Quantification and analysis of RSSH modifications in AtATO, AtCC1, AtCC1-like, and AtRSZ22A proteins. (Above: results of RSSH modifications; below: grayscale analysis of Western blot bands; CK: 0 μM H_2_S treatment; H_2_S: 200 μM H_2_S treatment. Data are presented as mean ± standard error (SE), from three independent experimental replicates (^*^P < 0.05, Student’s *t*-test).

We performed prokaryotic expression purification and initial screening of *in vitro* RSSH modifications. In total, seven genes from *Arabidopsis* were cloned: *AtATO* (1515 bp), *AtCC1* (1263 bp), *AtCC1*-like (1584 bp), *AtRSZ22A* (591 bp), *AtCBP80* (2547 bp), *AtSWAP* (2358 bp), and *AtMOS4* (762 bp). Nine prokaryotic expression vectors were constructed, namely, pCold-*AtATO*, pCold-*AtCC1*, pCold-*AtCC1*-like, pCold-*AtRSZ22A*, pCold-*AtCBP80*, pCold-*AtSWAP*, pCold-*AtMOS4*, XF245-*AtCC1*, and XF245-*AtCC1*-like*.* Due to purification difficulties with pCold-*AtCC1* and pCold-*AtCC1*-like, the XF245 vector was reconstructed for expression and purification, as shown in Supplementary Fig. 1 (all supplementary figures and tables can be accessed as online Supplementary Material). The primers used are listed in Supplementary Table 1. An enhanced biotin switch assay was used to demonstrate H_2_S-induced RSSH modifications *in vitro* for four of the seven studied proteins, namely, AtATO, AtCC1, AtCC1-like, and AtRSZ22A (Fig. 2). No significant changes in RSSH signals were detected for AtCBP80, AtSWAP, and AtMOS4 (data not shown).

### H_2_S mediated the RSSH modification of SF BraATO2 in *B. rapa*

Based on the results of the RSSH of candidate SFs from *Arabidopsis*, we chose AtATO as the focus for analysis. We further identified a highly homologous *B. rapa* gene, *BraATO2* (*Bra028740*), from the *B. rapa* database (http://brassicadb.cn). *BraATO2* consists of 1521 base pairs and encodes a protein comprised of 477 amino acids, which includes four potential Cys RSSH modification sites at Cys335, Cys413, Cys416, and Cys442 (Supplementary Fig. 2).

After constructing the recombinant plasmid pColdTF-*BraATO2*, we confirmed its validity through double-enzyme digestion (Supplementary Fig. 3A) and sequencing. The fusion protein was then expressed and purified *in vitro* (Supplementary Fig. 3B), and its RSSH levels were determined. Previous studies have shown that the trigger Factor (TF) protein, which was extracted and labeled from the pColdTF vector, dose not undergo RSSH modification [8]. Subsequently, the purified fusion protein was treated with H_2_S, and the resulting signal levels were analyzed using Western blotting combined with the biotin switch assay (Supplementary Fig. 3C). Exposure to 200 μM H_2_S significantly increased the RSSH intensity of BraATO2 compared with the control group, indicating that the SF BraATO2 undergoes H_2_S-mediated RSSH modification.

**Figure 3:**
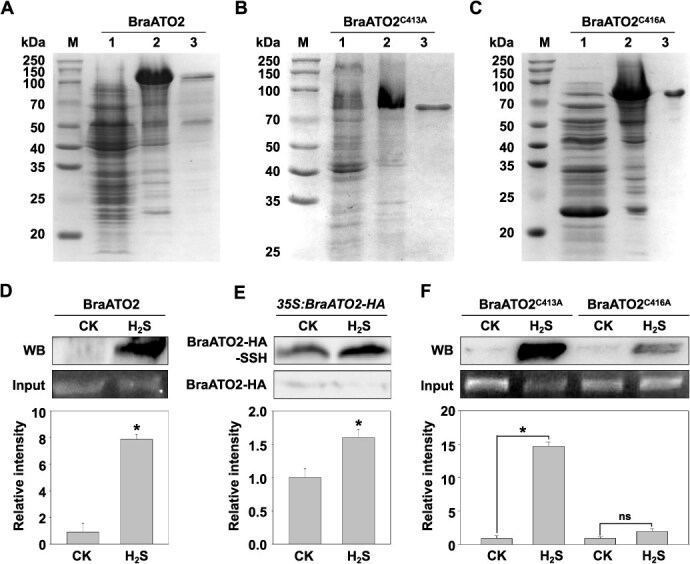
Detection of RSSH of BraATO2 in *Brassica rapa and Arabidopsis thaliana*. Induction and purification of the pCold-*BraATO2* fusion protein (A) and Cys site-mutated pET-28a-*BraATO2* fusion proteins (B, C) (M, protein molecular weight marker; 1, proteins extracted from uninduced *Escherichia coli*; 2, proteins extracted from IPTG-induced *E. coli*; 3, purified protein). (D) Detection and quantitative analysis of BraATO2 RSSH levels *in vitro*. (E) Detection and quantitative analysis of BraATO2 RSSH levels *in vivo* in *Arabidopsis* plants overexpressing *BraATO2* (BraATO2-HA-SSH: RSSH level of the tagged BraATO2-HA; BraATO2-HA: load control detected using a hemagglutinin tag (HA) antibody). (F) Detection and quantitative analysis of Cys site-mutated BraATO2 RSSH levels *in vitro*. CK: 0 μM H_2_S treatment; H_2_S: 200 μM H_2_S treatment. Data are expressed as the mean ± standard error (SE) of three independent experimental replicates (^*^P < 0.05, Student’s *t*-test; ns, no significant difference).

The primary targets of H_2_S RSSH modification are the active Cys residues, particularly those located within the zinc finger domains of C2H2 zinc finger proteins (https://zf.princeton.edu/). By aligning the homologous gene sequences of *A. thaliana* and *B. rapa* and using an online Cys site prediction tool (http://pcysmod.omicsbio.info/), we identified two Cys residues, Cys413 and Cys416, as potential modification sites within the zinc finger domain (Supplementary Fig. 4). Subsequently, these two Cys residues were mutated to Ala via site-directed mutagenesis. We successfully constructed expression vectors for the mutant proteins and verified them using enzyme digestion (Supplementary Fig. 3D) and sequencing. We expressed and purified the fusion proteins, followed by the measurement of their RSSH levels *in vitro* (Fig. 3A–C). Compared with BraATO2, the RSSH signal intensity of BraATO2^C416A^ was significantly reduced after treatment with 200 μM H_2_S. In contrast, the RSSH signal intensity of BraATO2^C413A^ remained unchanged after treatment with 200 μM H_2_S compared with the control (Fig. 3D and F). These findings indicate that Cys416 of BraATO2 is the primary site for H_2_S-mediated RSSH modification.

To further validate the findings *in vivo*, we examined the effect of H_2_S on the RSSH levels of BraATO2 in *Arabidopsis* overexpressing *BraATO2*. H_2_S treatment significantly increased BraATO2 RSSH levels in *Arabidopsis* overexpressing *BraATO2* (Fig. 3E), further confirming that BraATO2 serves as a direct substrate for H_2_S signaling.

### Alterations in the alternative splicing patterns of multiple genes in *BraATO2*-silenced strains

**Figure 4:**
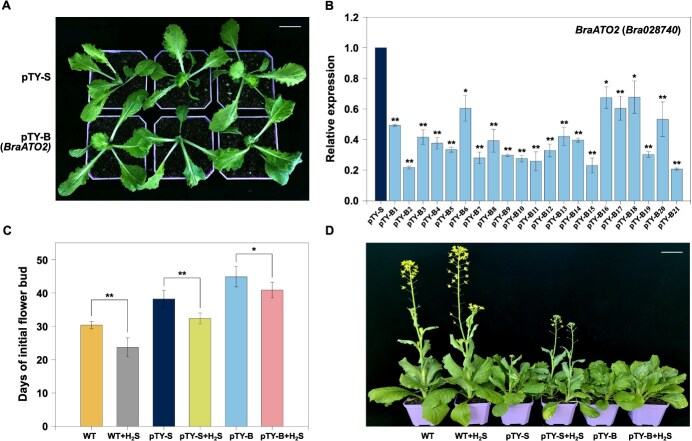
Identification of *BraATO2*-silenced *Brassica rapa* plants and analysis of H_2_S effects on their flowering. (A) Phenotypic comparison of VIGS-treated (pTY-*BraATO2*) and control (pTY-S) *B. rapa* plants after 2 weeks of gene silencing (scale bars = 5 cm). (B) Detection of *BraATO2* expression in various gene-silenced *B. rapa* plants. (C) Statistical analysis of initial flower bud days across various *B. rapa* lines. (D) Comparison of H_2_S sensitivity among various *B. rapa* lines in flowering time (scale bars = 9.0 cm). pTY-S: *B. rapa* plants treated with the empty vector pTY-S; pTY-B (1–21): 21 gene-silenced *B. rapa* plants treated with pTY-*BraATO2*. Data are expressed as the mean ± standard error (SE) of three independent experimental replicates (^*^P < 0.05, ^**^P < 0.01; Student’s *t*-test).

To investigate the *in vivo* function of *BraATO2*, we used the virus-induced gene silencing (VIGS) method to infect wild-type (WT) *B. rapa* seedlings. Observations were made 2 to 3 weeks post-treatment (Fig. 4A). *BraATO2* expression was quantified using real-time qPCR, with plants infected with the empty vector (pTY-S) serving as controls. The primers are listed in Supplementary Table 2. The VIGS method was highly effective. *BraATO2* expression levels significantly decreased in the 21 infected plants (Fig. 4B), especially Plants 2, 15, and 21, which exhibited the most effective silencing. Therefore, these three plants were selected as the silenced lines for subsequent AS analysis. The positive plants underwent statistical analysis to assess their flowering time.

The results showed that, in wild-type (WT) plants, H_2_S exhibits a promoting effect on flowering. After pTY-S treatment, the plants displayed delayed flowering (Fig. 4C). However, H_2_S treatment retained its ability to promote flowering. In VIGS-induced *BraATO2-*silenced plants, the flowering-promoting effect of H_2_S was significantly weakened. Nevertheless, the silencing of *BraATO2* does not completely block the flowering-promotion effect of H_2_S (Fig. 4D).

BraATO2 functions as an SF within the U2 spliceosome. Based on previous findings, we hypothesized that H_2_S-mediated RSSH modification may affect the function of BraATO2 in the spliceosome, resulting in changes in the AS patterns of specific downstream genes. We verified this hypothesis by analyzing the AS patterns of downstream genes in *BraATO2*-silenced plants treated with exogenous H_2_S, which were generated using VIGS. By reviewing the literature and screening the *B. rapa* transcriptome database (Supplementary Fig. 5 and Supplementary Tables 3–5), the following six genes were identified as potential candidate: *BraAGL31*/*MAF2* (*Bra031888*), *BraGRP8* (*Bra011869*), *BraFER1* (*Bra005677*), *BraFER3* (*Bra003226*), *BraARF1* (*Bra012376*), and *BraLOG1* (*Bra034399*). We analyzed the AS events and sites in these six candidate downstream genes. The primers used in this study are listed in Supplementary Table 6.

**Figure 5:**
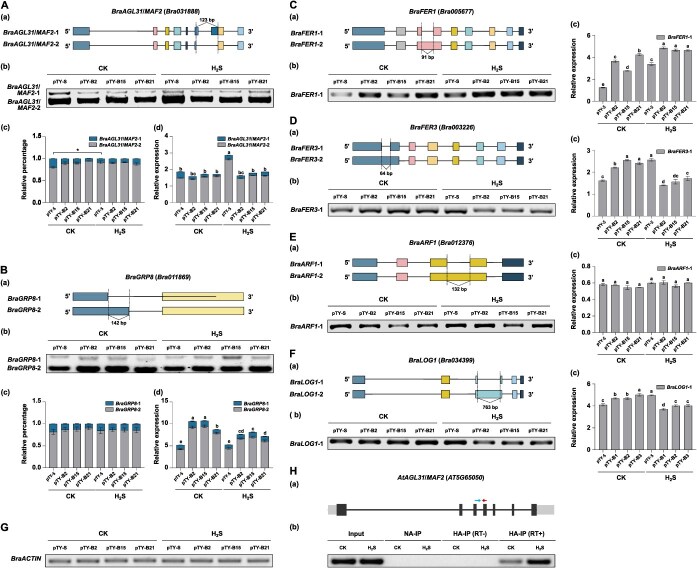
Effects of H_2_S-mediated BraATO2 RSSH modification on downstream gene alternative splicing (AS) patterns. (A) Analysis of AS patterns of *BraAGL31/MAF2* in plants treated with the empty vector (pTY-S) and *BraATO2*-silenced plants (pTY-B2/15/21) under H_2_S treatment (a: Schematic diagram of AS events in *BraAGL31*/*MAF2*; b: Detection by polymerase chain reaction (PCR) of changes in AS patterns in two *BraAGL31*/*MAF2* transcripts; c: Proportion analysis of two *BraAGL31*/*MAF2* transcripts; d: Relative expression levels of two *BraAGL31*/*MAF2* transcripts). CK: 0 μM H_2_S treatment; H_2_S: 200 μM H_2_S treatment. Data are expressed as the mean ± standard error (SE) of three independent experimental replicates (^*^P < 0.05, Student’s *t*-test; distinct lowercase letters indicate significant differences at *P* < 0.05, Tukey’s Honestly Significant Difference test). (B) Analysis of AS patterns of *BraGRP8* in pTY-S and pTY-B2/15/21 plants under H_2_S treatment (a: Schematic diagram of AS events in *BraGRP8*; b: Detection by PCR of changes in AS patterns in two *BraGRP8* transcripts; c: Proportion analysis of two *BraGRP8* transcripts; d: Relative expression levels of two *BraGRP8* transcripts). (C) Analysis of AS patterns of *BraFER1* in pTY-S and pTY-B2/15/21 plants under H_2_S treatment (a: Schematic diagram of AS events in *BraFER1*; b: Detection by PCR of changes in AS patterns in *BraFER1*; c: Relative expression levels of the ×1 transcript of *BraFER1*). (D) Analysis of AS patterns of *BraFER3* in pTY-S and pTY-B2/15/21 plants under H_2_S treatment (a: Schematic diagram of AS events in *BraFER3*; b: Detection by PCR of changes in AS patterns in *BraFER3*; c: Relative expression levels of the ×1 transcript of *BraFER3*). (E) Analysis of AS patterns of *BraARF1* in pTY-S and pTY-B2/15/21 plants under H_2_S treatment (a: Schematic diagram of AS events in *BraARF1*; b: Detection by PCR of changes in AS patterns in *BraARF1*; c: Relative expression levels of the ×1 transcript of *BraARF1*). (F) Analysis of AS patterns of *BraLOG1* in pTY-S and pTY-B2/15/21 plants under H_2_S treatment (a: Schematic diagram of AS events in *BraLOG1*; b: Detection by PCR of changes in AS patterns in *BraLOG1*; c: Relative expression levels of the ×1 transcript of *BraLOG1*). (G) Expression analysis of the control gene *BraACTIN* in this experiment. (H) RNA immunoprecipitation (RNA-IP) analysis conducted to examine the regulatory role of H_2_S in the binding ability of BraATO2 to the pre-mRNA of the flowering-related gene *AtAGL31*/*MAF2* (a: Shows the binding site of BraATO2-HA for the pre-mRNA of *AtAGL31*/*MAF2*. The arrows indicate the upstream and downstream primers. The primer sequences are listed in Supplementary Table 6; b: Results of RNA-IP detection for *Arabidopsis* overexpressing *BraATO2* with H_2_S treatment). CK: 0 μM H_2_S treatment; H_2_S: 200 μM H_2_S treatment. Input: Positive control (No IP); NA-IP: No antibody was used during immunoprecipitation as a negative control; HA-IP (RT+) or HA-IP (RT-): PCR was performed following RNA-IP against HA with (RT+) or without (RT-) reverse transcription.

Transcriptomic analysis revealed that H_2_S exposure of plants treated with pTY-S induced exon skipping in *BraAGL31*/*MAF2* (Fig. 5A-a). Following H_2_S treatment, the proportion of the *BraAGL31*/*MAF2–1* transcript decreased significantly in pTY-S plants, along with an increased proportion of the *BraAGL31*/*MAF2-2* transcript. Conversely, *B. rapa* lines with silenced *BraATO2* expression exhibited no changes in the proportions of *BraAGL31*/*MAF2–1* and *BraAGL31*/*MAF2-2* transcripts after H_2_S treatment (Fig. 5A-b). The quantitative data shown in Fig. 5A-c further support this finding. Quantitative analysis of the total expression levels of the two transcripts of *BraAGL31*/*MAF2* indicated that H_2_S treatment significantly increased the total expression levels of the two *BraAGL31*/*MAF2* transcripts in the pTY-S plants. However, the total expression levels in the silenced lines did not increase significantly (Fig. 5A-d). The silencing of SF BraATO2 in *B. rapa* indicates the weakened effect of H_2_S on the AS pattern of *BraAGL31*/*MAF2*. In *Arabidopsis*, *AtAGL31*/*MAF2* (an *FLC*-like gene) and the *FLC* cluster are paralogous due to a larger chromosomal duplication event [32, 33]. H_2_S modulates the AS pattern of the flowering time regulator *BraAGL31*/*MAF2*, thereby altering the proportions of its various transcripts, which may play distinct roles in regulating plant flowering time. Although the functional differences between the two specific transcripts of *BraAGL31*/*MAF2* were unclear, our findings indicate a direct correlation between H_2_S signaling and the regulation of plant flowering time via AS. Further studies exploring this in the future are warranted.

Additionally, the transcriptomic analysis indicated that H_2_S can cause intron retention in *BraGRP8* (Fig. 5B-a). However, reverse transcription (RT)-PCR analysis showed no significant changes in the ratios of the two *BraGRP8* transcripts in both pTY-S and pTY*-BraATO2* lines after H_2_S treatment (Fig. 5B-b), and their AS patterns remained unaffected (Fig. 5B-c). This indicates that *BraGRP8* is not a downstream target of the H_2_S RSSH-modified SF BraATO2. Nevertheless, the total expression level of the *BraGRP8* significantly decreased after H_2_S treatment (Fig. 5B-d), indicating that H_2_S plays a regulatory role in *BraGRP8* expression, although the mechanism remains unclear. Four additional genes, *BraFER1*, *BraFER3*, *BraARF1*, and *BraLOG1*, exhibited AS events in the preliminary transcriptome data (Fig. 5C-a to F-a). Furthermore, significant changes in the total expression of *BraFER1*, *BraFER3*, and *BraLOG1* were observed in both pTY-S and pTY*-BraATO2* lines following H₂S treatment (Fig. 5C-c, D-c, and  F-c). However, AS of these genes was not detected on RT-PCR analysis (Fig. 5C-b to F-b). Consequently, the association of these four genes with BraATO2 could not be confirmed at this stage. Several factors might contribute to this, the most significant of which would be the sensitivity of RT-PCR technology in detecting AS transcripts with different expression levels. Furthermore, the limitations of the VIGS method restrict the experimental material to 5-week-old plant leaves, whereas the transcriptome database employs 2-week-old seedlings for detection.

To investigate whether H_2_S regulates AS patterns of flowering genes through BraATO2, we conducted RNA immunoprecipitation (RNA-IP) experiments in *Arabidopsis* plants overexpressing *BraATO2* (tagged with HA). These experiments assessed the binding affinity of BraATO2 with *AtAGL31*/*MAF2*, a homologous gene of *BraAGL31*/*MAF2* in *B. rapa*, following exposure to H_2_S. Treatment with H_2_S significantly enhanced the binding capacity of BraATO2 with the *AtAGL31*/*MAF2* pre-mRNA (Fig. 5H). These findings, along with the experimental results shown in Fig. 3, further confirm that H_2_S modifies the binding ability of BraATO2 with downstream target genes through RSSH, thereby regulating the AS pattern of flowering genes.

**Figure 6:**
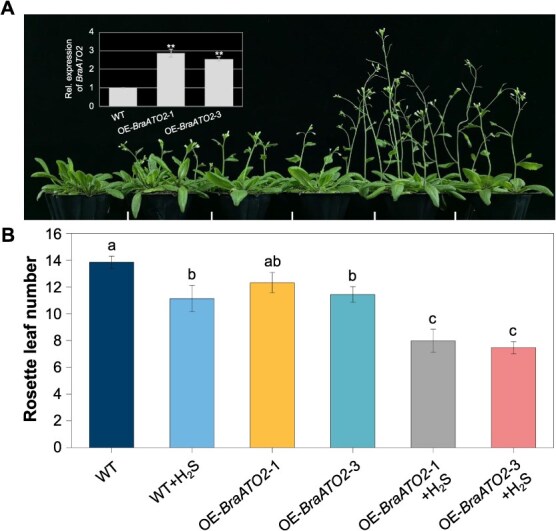
Response of *Arabidopsis* flowering to H_2_S treatment after heterologous overexpression of *BraATO2*. (A) Response of OE-*BraATO2* lines to H_2_S treatment during the flowering process of *Arabidopsis* (scale bars = 1.5 cm). OE-*BraATO2*–1 and OE-*BraATO2*–3 represent two overexpression lines. The bar chart in the upper left corner of panel A shows the expression levels of *BraATO2* in the overexpressing plants. Reverse transcription quantitative polymerase chain reaction was performed to analyze *BraATO2* expression in the transgenic lines. All expressions were normalized using *AtACTIN2* as the internal control. (B) Statistics on rosette leaf number at flowering for different *Arabidopsis* lines under different treatments. WT: wild-type plants; H_2_S: 100 μM treatment. Data are expressed as the mean ± standard error (SE) of three independent experimental replicates (^**^P < 0.01, Student’s *t*-test; distinct lowercase letters indicate significant differences at *P* < 0.05, Tukey’s HSD test; 10–15 for each replicate).

Based on the above results, we conclude that BraATO2 is a direct target protein for H_2_S signaling through RSSH. In this pathway model, H_2_S can influence BraATO2 function via RSSH modification, thereby regulating the AS patterns of downstream genes and, ultimately, plant flowering. Assuming that this pathway exists, the heterologous expression of *BraATO2* in *Arabidopsis* should theoretically alter its sensitivity to H_2_S treatment. To validate this hypothesis, we overexpressed *BraATO2* driven by the 35S promoter in *Arabidopsis* and exposed positive plants to H_2_S. The two overexpression lines, OE-*BraATO2*–1 and OE-*BraATO2*–3, exhibited increased sensitivity to H_2_S treatment compared with wild-type plants, resulting in significantly earlier flowering times. Furthermore, under normal growth conditions without H_2_S treatment, some OE-*BraATO2* lines flowered earlier than wild-type plants (Fig. 6).

## Discussion

**Figure 7:**
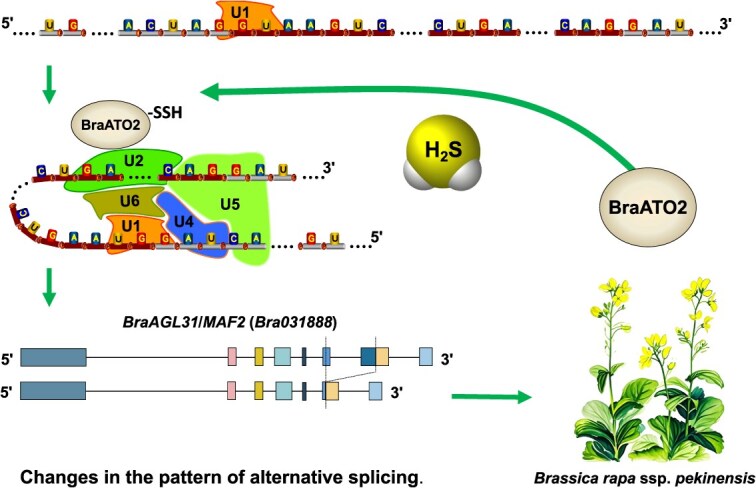
Schematic diagram of how H_2_S influences flowering gene AS patterns through RSSH modification of BraATO2 to promote plant flowering.

Flowering is a key developmental transition that shifts plants from the vegetative to the reproductive growth stages. Significant insights have been gained in *Arabidopsis* regarding the molecular mechanisms regulating flowering time. Based on the plant response to environmental conditions, such as light and temperature, during the flowering process, the regulation of flowering is generally categorized into the vernalization, autonomous, photoperiod, and gibberellin pathways [34, 35]. However, many pathways still require further in-depth analysis. This study found that the gasotransmitter H_2_S can RSSH modify the SF BraATO2 at Cys416 in *B. rapa* (Fig. 3F). RSSH modification can induce changes in the AS pattern of its downstream key flowering gene, *BraAGL31*/*MAF2*, at the transcription level (Fig. 5A), ultimately inducing an earlier flowering time for the plant (Fig. 1A). This discovery proposes BraATO2 as a novel direct target for H_2_S and also reveals a new regulatory pathway for flowering in *B. rapa* (Fig. 7). Furthermore, the study provides novel insights that would guide the future development of plant flowering regulator. Naturally, the complexity of plant flowering regulation and gene action networks indicates the involvement of other related mechanisms. The regulation of plant flowering by H_2_S is only partially dependent on BraATO2 (Fig. 4C and D). This may be due to the silencing efficiency of VIGS or, alternatively, H_2_S may promote flowering through alternative pathways. A comprehensive analysis of the mechanisms underlying this process still requires further exploration.

The genomes of cruciferous crops, such as *B. rapa* ssp. *chinensis* and *B.rapa* ssp. *pekinensis*, display a clear syntenic relationship with the *A. thaliana* genome, which facilitates molecular biological research on these crops. The *Arabidopsis FLOWERING LOCUS C* (*FLC*) serves as a crucial floral repressor in maintaining the vernalization response. *MADS AFFECTING FLOWERING 2* (*MAF2*, also referred to as *AGL31*), *MAF3, MAF4,* and *MAF5* are four *FLC* paralogs arranged in a tandem array on the bottom of *Arabidopsis* chromosome V. *MAF2* functions as a floral repressor [36]. In *Arabidopsis*, the SHORT VEGETATIVE PHASE (SVP) is essential for the ambient temperature-responsive pathway. SVP forms complexes with MAF2 to bind to DNA. At low temperatures, the predominant *MAF2* splice variant encodes a protein that interacts with SVP, thereby repressing flowering. At high temperatures, another splice isoform of MAF2, stimulates the introduction of a premature termination codon. In *B. rapa* ssp. *chinensis*, which is genetically similar to *B. rapa* ssp. *pekinensis*, *BcMAF2* plays a critical role in flowering [37]. Furthermore, the similarity in the alternative splicing patterns between *BraAGL31*/*MAF2* detected in our study and those reported in *Arabidopsis* proves that *BraAGL31*/*MAF2* is an essential flowering regulatory gene in *B. rapa* ssp. *pekinensis,* with its AS pattern significantly influencing flowering regulation.

Our previous studies have demonstrated that H_2_S mediates the RSSH of various homologous genes of *BraFLC* in Chinese cabbage, thereby influencing the binding of these transcription factors to their downstream target genes and leading to early flowering [29]; Xuan *et al*. [38] reported that H_2_S promotes gibberellin synthesis, thereby stimulating plant flowering. In the current study, we found that H_2_S influences the function of BraATO2 via RSSH modification, ultimately altering the AS pattern of downstream genes and subsequently influencing flowering time. These seemingly fragmented research findings currently cannot be integrated into a single pathway model. Perhaps, as more reports and information emerge, the interrelationships among them will be elucidated. A similar situation has been observed in the research on the regulation of ABA signaling by H_2_S. Hu *et al*. [39] reported that H_2_S signaling leads to RSSH of amino acid residues C115 and C118 in ERF.D3, activating the expression of *CYP707A2*, which encodes for an ABA-degrading enzyme, thereby promoting ABA degradation. Chen *et al*. [40] found that H_2_S induces the RSSH of two sites, Cys131 and Cys137, on SnRK2.6, enhancing ABA signal transduction and promoting ABA-induced stomatal closure. Zhou *et al*. [21] indicated that H_2_S induced the RSSH of Cys250 in ABI4, further promoting the transcriptional activation of MAPKKK18. Given the limitations of currently available research methods, scientific inquiry often resembles the fable of the blind men and the elephant. However, comprehensive understanding often begins with the accumulation of local insights. Research on the physiological functions of H_2_S in plants, especially in understanding RSSH targets, is still in the preliminary stages. As these fragmentary research findings accumulate, we anticipate to eventually achieve comprehensive understanding of the mechanisms.

SF ATO has been reported to encode a protein similar to SF3a60 [41], exhibiting the highest potential for regulating plant flowering. Therefore, we subsequently analyzed BraATO2 as to explore the potential role of H_2_S in modulating flowering in *B. rapa* via this protein. The experimental results confirmed that H_2_S modulated the function of BraATO2 in *B. rapa* through RSSH modification, affecting the AS pattern of the downstream flowering gene *BraAGL31*/*MAF2* and thus regulating plant flowering (Fig. 7). Various direct targets of H_2_S in plants have been reported, such as ACO, BrRuBisCO, DES, ABI4, SnKR2.6, BraFLC, and U2AF65a. Here we confirmed that BraATO2 is a direct target of H_2_S-mediated RSSH modification, enhancing the understanding of the underlying mechanisms by which H_2_S functions as a gasotransmitter.

H_2_S has various irreplaceable physiological functions [16, 42]; however, why H_2_S exhibits such a wide range of functional effects remains a perplexing question. Considering the abundance of proteins with potentially reactive sites within cells, the RSSH modification by H_2_S of Cys residues in target proteins may be a key factor. Nevertheless, the specificity exhibited by these RSSH-modified Cys residue sites remains to be elucidated.

**Figure 8:**
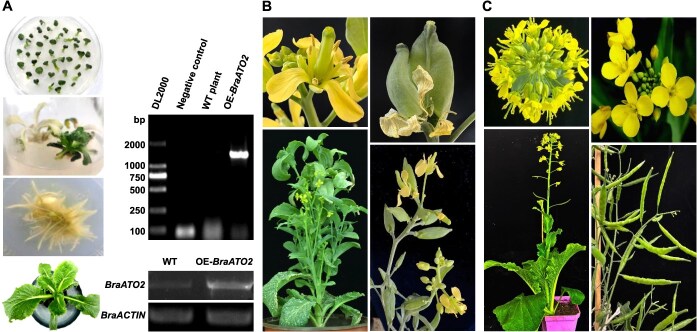
Genetic transformation of *BraATO2* in wild-type (WT) *B. rapa*. (A) Left column (top to bottom): differentiation of regenerated buds, induction of roots, rooting process, transplantation of regenerated plants into soil after 30 days. Top right column: DNA identification post-genetic transformation. Bottom right column: Expression analysis of *BraATO2* post-genetic transformation. (B) Phenotypic observation of OE-*BraATO2 B. rapa*. (C) Phenotypes of WT *B. rapa*.

To provide more genetic evidence for the function of BraATO2, we genetically transformed *B. rapa* to create *BraATO2* overexpression lines and conducted phenotypic assessments. Despite the significant challenges associated with the genetic transformation of *B. rapa*, we conducted numerous attempts but ultimately obtained only one overexpression plant. Although the quantity of plants is insufficient to provide definitive phenotypic evidence, we still hope that the phenotype of this plant can offer some reference for subsequent research. Observations revealed that the floral organs of OE-*BraATO2* plant exhibited abnormal development compared with the WT *B. rapa*, as shown in Fig. 8. OE-*BraATO2* exhibited short, thick stigmas, early stigma exposure, and petal-like stamen formation, resulting in flowers with five petals. Male plants were impaired in their ability to produce normal pollen, leading to seed abortion and abnormal silique development. The emergence of these phenotypes does not exclude the possibility of growth and developmental abnormalities resulting from the plant tissue culture process, necessitating further investigation and monitoring.

## Conclusion

Our study revealed a novel pathway by which the gaseous signaling molecule H_2_S promotes flowering in *B. rapa*. Specifically, H_2_S alters the splicing factor BraATO2, a key component in gene-selective splicing in *B. rapa,* via RSSH. H_2_S regulates BraATO2 function by modifying the Cys residue located at position 416. This regulation leads to altered patterns of gene-specific splicing, particularly in the *FLC-*like gene family member *BraAGL31*/*MAF2* (Fig. 7). Consequently, this mechanism facilitates flowering in *B. rapa*. Our findings not only identify a novel direct target of H_2_S signaling but also shed light on a new mechanism underlying flowering regulation in *B. rapa*, providing valuable insights for the development of innovative plant flowering regulators.

## Materials and methods

### Cultivation of materials

This study utilized two experimental plant materials: *A. thaliana* and *B. rapa*. The *Arabidopsis* plants used in this study comprised the WT Columbia-0 strain and a double mutant, *lcd/des1,* with the deletions in the genes encoding the primary endogenous H_2_S-producing enzymes, derived from the Columbia genetic background. The double mutant *lcd/des1* was created by hybridizing the T-DNA insertion mutants *lcd* (SALK-082099) and *des1* (SALK205358C), generously provided by Professor Shaowu Xue of Huazhong Agricultural University.

The overexpression line of *BraATO2* in *Arabidopsis* was obtained via Agrobacterium-mediated transformation using the 35S promoter, and a homozygous line was established in the T3 generation. The over expression vector used was XF350, which was modified from the basic frame of pCAMBIA2302 and contained an HA tag, generously provided by Professor Xiaofeng Cao from the Institute of Genetics and Developmental Biology, Chinese Academy of Sciences (CAS). The seeds were planted in a substrate with a 1:1:1 ratio of soil, perlite, and vermiculite. The cultivation conditions were 23°C, 60% relative humidity, a 16/8-h light/dark cycle, and a light intensity of 3000 lux.

The inbred lines, “Shannong 208” and “Zhongbai 60” of *B. rapa* were used, kindly supplied by the Shanxi Academy of Agricultural Sciences. The seeds were uniformly sown on three layers of moist gauze and underwent a 30-day dark treatment at 4°C to induce vernalization. Subsequently, the seeds were transferred to a nutrient medium and cultivated for an additional 14 days. Once two true leaves emerged, VIGS was performed using the gene gun technique. Three weeks’ post-transformation, the silenced plants were identified within the *B*. *rapa* population. The cultivation conditions for *B. rapa* and *A*. *thaliana* were identical.

For the VIGS method. The WT plants, 14 days old and vernalized, were subjected to gene gun bombardment, followed by a dark incubation for 24 hours before being transferred to normal growth conditions (22–23°C, relative humidity 60%, a light intensity of 3000 lux, 16/8-h light/dark cycle).

### Treatment of the materials

In this study, sodium hydrosulfide (NaHS), an H_2_S donor, was used as the treatment agent. Treatment commenced when *B. rapa* reached the 7-day growth stage and developed heart leaves. Specifically, a 100-μM H_2_S was sprayed daily onto the leaves of *B. rapa*, whereas the control group received distilled water. The spraying was conducted daily at a consistent time and volume until the *B. rapa* reached the flowering stage.

### Prokaryotic expression and protein purification

To express the target protein in *E. coli,* the mature coding sequence of the target gene was first cloned into an expression vector. In this study, three prokaryotic expression vectors were used: pCold-TF, pET-28a(+)-sumo and XF-245 provided by Professor Xiaofeng Cao from CAS. After positive identification through double restriction enzyme digestion, the recombinant plasmids were successfully transformed into *E. coli* BL21(DE3) for inducible expression of the exogenous protein. The recombinant protein was then purified using Ni-NTA Sefinose™ Resin (Sangon Biotech, Shanghai, China), according to the manufacturer's instructions. Finally, the purity of the protein was confirmed by 10% SDS-PAGE electrophoresis.

After induction with 0.3 mM IPTG at 15°C, significant expression of the pColdTF-*BraATO2* fusion protein was detected in both whole cells and supernatants. Purification with 50 mM imidazole yielded a relatively pure protein (Supplementary Fig. 3B). This protein was then used to evaluate the levels of RSSH modification (Fig. 3D).

After induction with 0.7 mM IPTG at 28°C, significant expression of both pET-28a(+)-sumo-*BraATO2*^C413A^ and pET-28a(+)-sumo-*BraATO2*^C416A^ fusion proteins was observed in whole cells and supernatants. Purification with 150 mM imidazole resulted in a relatively pure protein (Fig. 3B and C). Subsequently, the purified protein solution was utilized to quantify the levels of RSSH modification (Fig. 3F).

### Biotin switch analysis of RSSH modifications *in vitro* and *in vivo*

The purified proteins treated with H_2_S underwent RSSH modification analysis *in vitro* via an improved biotin switch method, as outlined in reference [17]. The main steps of the experimental process are as follows. H_2_S Treatment: Purifying the target protein via nickel affinity chromatography, followed by incubation with 200 μM H_2_S at 4°C for 30 minutes (the control group received an equal volume of ddH_2_O); Protein Precipitation: Proteins were precipitated with pre-cooled reagents and dissolved in 100 μl of HEN buffer; Blocking Free Sulfhydryl Groups: 200 μl of methyl methanethiosulfonate (MMTS) was added to the solution and incubated at 50°C for 25 minutes to block free sulfhydryl groups; Protein Concentration and Biotin Labeling: Proteins were precipitated again and dissolved in 100 μl of HEN buffer containing 1% SDS and 30 μl biotin-HDPD [N-(6- (biotinamido) hexyl)-3′-(2′-pyridyldithio)-propionamide] solution. The mixture was then incubated on a rotator at 25°C for 3 hours; Western Blotting: Samples were separated by SDS-PAGE and transferred to a nitrocellulose membrane. Western blot analysis was performed using a biotin antibody, followed by alkaline phosphatase-labeled goat antimouse IgG and NBT/BCIP color detection. The obtained data were analyzed using Image J software (NIH, Bethesda, MD, USA) for grayscale quantification.

For *in vivo* experiments, 12-day-old overexpressing plants (*35S:BraATO2-HA*) were used as materials, and treated with 200 μM H_2_S for 60 minutes; total proteins were extracted using a protein extraction buffer (25 mM Tris, 100 mM NaCl, 0.2% [v/v] Triton X-100 [pH 8.0]); free thiols in the total proteins were blocked with MMTS at 50°C for 25 minutes; then proteins were precipitated and then resuspended in HEN buffer containing 1% SDS; 4 mM Biotin-HPDP was added to label the persulfidated proteins (the MMTS blocking and Biotin-HPDP labeling procedures were the same as described in the above *in vitro* experiment); 60 ml of streptavidin-agarose beads (Sigma-Aldrich, St. Louis, MO, USA) were added, and the biotinylated proteins were purified by overnight immunoprecipitation at 4°C; biotinylated proteins were separated by SDS-PAGE and transferred onto a NC membrane, followed by Western blot analysis of the RSSH levels of the labeled proteins using HA antibody (Abmart, Shanghai, China), diluted 1:3000. See our published literature for details [43].

### Gene silencing induced by the pTY-*BraATO2* virus

A 40-nucleotide (nt) fragment with high specificity to the open reading frame of the *BraATO2* gene, and a 5′ end of NTAG, NTAA, or NTGA (where N = A, T, or C), was chosen. Subsequently, a corresponding 80-nt palindromic DNA fragment was designed and synthesized. Homologous sequences of 15 nt, matching the linearized vector (pTY-S, provided by Prof. Ying Li of Nanjing Agricultural University), were inserted at the 3′ and 5′ ends, respectively. The 110-nt DNA fragment was synthesized via homologous recombination [44] with the linearized vector at a 3:1 ratio. The primers and the pTY-*BraATO2* recombinant plasmid were synthesized by Genscript biotechnology company of Nanjing. Then, the recombinant plasmid underwent transformation, extraction, detection, purification, and concentration. Once the concentration reached 2 mg/ml, the gene gun was utilized for the particle bombardment-mediated transformation. The *B. rapa* seedlings were transplanted into the soil and grown for 2 weeks until the emergence of two true leaves, making the optimal stage for achieving high transformation efficiency. Particle bombardment was performed using the PDS 1000/He biolistic delivery system (Bio-Rad, Hercules, CA, USA). The optimal bombardment parameters included a target distance of 9 cm, a helium pressure of 8.96 MPa, and a vacuum pressure of 0.36 MPa. Following bombardment, the plants were immediately transferred to a nutrient medium and kept in darkness for 24 hours. Afterward, the plants were incubated in a greenhouse for three weeks before identification [45].

### Genetic transformation of *B. rapa*

The vector employed in this study was XF350 (generously provided by Professor Xiaofeng Cao from CAS), which was modified from the basic backbone of pCAMBIA2302 by adding an HA tag. The strains used were *E. coli* DH5α and *Agrobacterium tumefaciens* EHA105*,* both stored in our laboratory.

The pCold-*BraATO2* recombinant plasmid served as a template for PCR amplification of genes with homologous arms using *pfu* high-fidelity DNA polymerase (TransGen, Beijing, China). The primer sequences are listed in Supplementary Table 2. The amplified product was then used to construct a eukaryotic expression vector. The construction process comprised PCR product recovery, plasmid extraction, linear vector acquisition, T5 homologous recombination ligation, transformation into *E. coli*, bacterial liquid PCR identification, and sequencing. The positive recombinant plasmid was subsequently employed for genetic transformation in *B. rapa*. The transformation process utilized a traditional tissue culture system, following the methodology described in reference [46]. The formula for the genetic transformation medium for *B. rapa* is detailed in Supplementary Table 7.

Exogenous overexpression of *BraATO2* in *Arabidopsis* was achieved using the aforementioned vector. Homozygous T3 generation lines of *A. thaliana* were obtained through Agrobacterium-mediated floral dip transformation and identified for further experiments.

### RNA extraction, cDNA synthesis, and gene expression analysis

Total RNA was extracted from the samples using TRIzol reagent (Invitrogen, California, USA). Reverse transcription was performed according to the manufacturer’s instruction, utilizing 5× All-In-One RT MasterMix (abm, Vancouver, Canada). ACTIN served as the reference gene, and the expression levels were quantified by using the 2^−ΔΔCT^ method. Real-time qPCR was conducted on the Bio-Rad CFX96 PCR detection system (Bio-Rad, Hercules, CA, USA). The primer sequences are provided in Supplementary Table 1.

### RNA immunoprecipitation (RNA-IP)

RNA-IP was performed following the protocol detailed by Ma *et al*. [30]. Twelve-day-old seedlings (2 g) were ground in liquid nitrogen and homogenized in extraction buffer (20 mM Tris–HCl, pH 7.5, 300 mM NaCl, 5 mM MgCl₂, 5 mM DTT, and 1% (v/v) protease inhibitor cocktail) at a 3:1 ratio (buffer to seedlings, v/w), the homogenate was centrifuged at 13 000 rpm for 10 minutes, three times, to pellet precipitates; then 40 μl of protein-G agarose (Solarbio, Beijing, China) were washed with PBS buffer and pelleted by centrifugation at 4°C for 5 minutes; the HA antibody (Abmart, Shanghai, China) diluted 1:200 was incubated with the sample for 3 hours at 4°C under rotation; protein-G agarose was added at a ratio of 1:50, and the incubation was extended for another 2 hours; the immune complexes were washed four to five times with 1.5 ml of extraction buffer (0.5% NP-40) and then twice with RNA-induced silencing complex (RISC) buffer (40 mM Hepes, pH 7.4, 100 mM KOAc, 5 mM MgOAc, 4 mM DTT). Finally, the immune complexes were resuspended in 100 μl of RISC buffer.

### Data statistical analysis

The data were organized with Excel 2019 software (Microsoft, Redmond, WA, USA) and statistically analyzed using SPSS19.0 (IBM, Chicago, IL, USA). Data are presented as mean ± SE of three independent experimental replicates. For *B. rapa*, 6 to 10 individual plants were measured per replicate, whereas for *A. thaliana*, 10 to 15 individual plants were measured per replicate. Significance was determined at *P < 0.05 using the Student's *t* test. Distinct lowercase letters indicate significant differences (*P* < 0.05) according to Tukey’s HSD test. The graphs were plotted using Origin 2019b (Electronic Arts Inc, CA, USA).

## Supplementary Material

Web_Material_uhaf190

## Data Availability

All relevant data can be found in the manuscript and supplementary materials.
