# Supplementary material for: H2S promotes flowering in Brassica rapa ssp. pekinensis by persulfidation of the splicing factor BraATO2
Source: Hortic Res. 2025 Jul 16;12(10):uhaf190. doi: 10.1093/hr/uhaf190 (PMC12539866; doi:10.1093/hr/uhaf190)
Supplement: Web_Material_uhaf190 [file web_material_uhaf190.zip › S1-S5â_"â_"7.14.pdf]

Supplementary Figure 1. Gene cloning and construction of prokaryotic expression vectors: pCold-*AtATO*, XF245-*AtCC1*, XF245-*AtCC1*-like, pCold-*AtRSZ22A*, pCold-*AtCBP80*, pCold-*AtSWAP*, and pCold-*AtMOS4*.

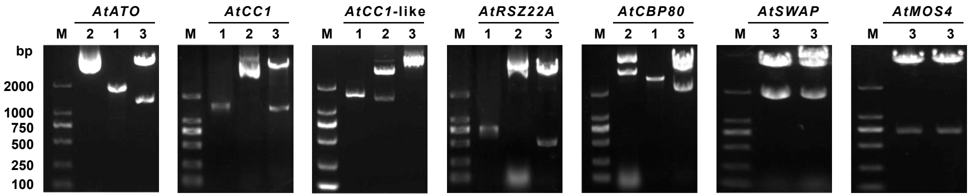

(M, DL2000 DNA marker; 1. Target gene PCR products; 2. Recombinant plasmid; 3. Double enzyme digestion products of the recombinant plasmid.).

ID	Position	Modification	FPR	Peptide
Bra005882	11	S-sulfhydration	34.91%	TLLDQIR C NHEEIER
Bra005882	173	S-sulfhydration	0.52%	SHPEKIP C KLKLSRQ
Bra005882	339	S-sulfhydration	3.97%	EAKVKKL C NLLDETI
Bra005882	419	S-sulfhydration	24.90%	GLGQKFK C EICRNKI
Bra005882	422	S-sulfhydration	5.39%	QKFKCEI C RNKIYKG
Bra005882	448	S-sulfhydration	28.62%	QHQDGMR C LGIPNTK

ID	Position	Modification	FPR	Peptide
Bra028740	335	S-sulfhydration	5.73%	EAKVKKL C SLLDETI
Bra028740	413	S-sulfhydration	19.19%	GLGQKFE C EICGNSV
Bra028740	416	S-sulfhydration	24.72%	QKFECEI C GNSVYMG
Bra028740	442	S-sulfhydration	44.97%	QHQHGMR C LGIPNTK

ID	Position	Modification	FPR	Peptide
AT5G06160	213	S-sulfhydration	43.03%	DRILSKV C SDFEEQY
AT5G06160	333	S-sulfhydration	3.81%	EAKVKKL C NLLDETI
AT5G06160	411	S-sulfhydration	66.00%	GLGQEFK C EICGNYS
AT5G06160	414	S-sulfhydration	53.86%	QEFKCEI C GNYSYWG
AT5G06160	440	S-sulfhydration	38.20%	RHQHGMR C LGIPNTK

Supplementary Figure 2. Prediction of cysteine sites potentially undergoing RSSH modification

Gene name	Cysteine site
Bra005882	11, 173, 339, 419, 422, 448
Bra028740	335, 413, 416, 442
AT5G06160	213, 333, 411, 414, 440

Cysteine cite domain prediction
(http://pcysmod.omicsbio.info/)

Supplementary Figure 3. Detection of RSSH modification of BraATO2 in *Brassica rapa*.

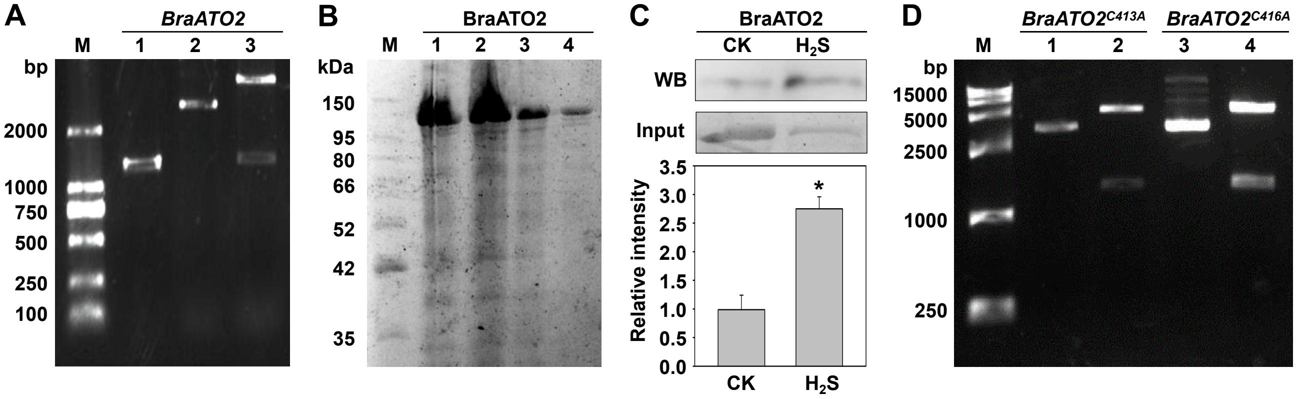

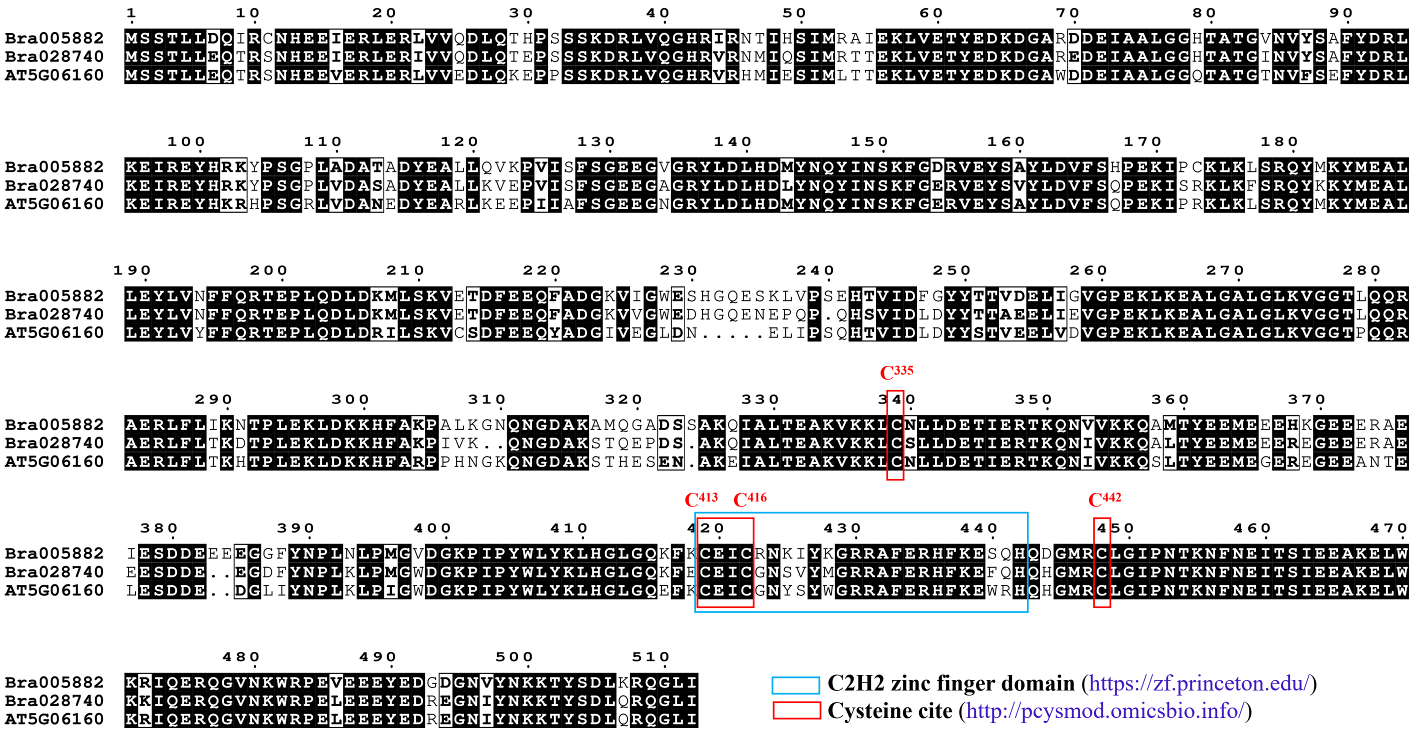

Supplementary Figure 4. Prediction of cysteine residues and C2H2 zinc finger domains within the conserved sequence of BraATO2.

Supplementary Figure 5. Heatmap depicting the correlation of gene expression levels between sample pairs.

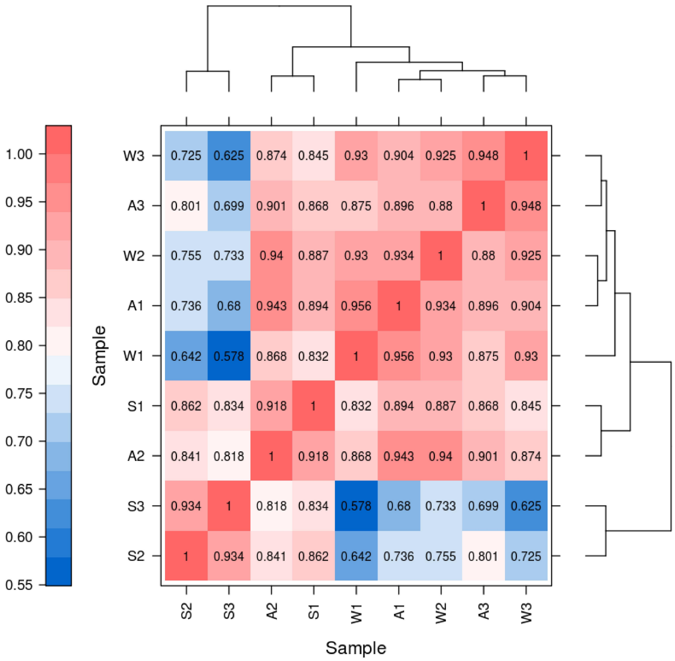

A, **S**, and **W** denote three distinct treatments applied to Chinese cabbage: **A** (HA, an inhibitor of H_2S synthesis), **S** (H_2S), and **W** (wild-type). Each treatment was biologically replicated threefold.
