# Supplementary material for: H2S promotes flowering in Brassica rapa ssp. pekinensis by persulfidation of the splicing factor BraATO2
Source: Hortic Res. 2025 Jul 16;12(10):uhaf190. doi: 10.1093/hr/uhaf190 (PMC12539866; doi:10.1093/hr/uhaf190)
Supplement: Web_Material_uhaf190 [file web_material_uhaf190.zip › Supplementary Tables 1-2-3-4-6-7.docx]

**Supplementary Table 1.** Primers for gene amplification and construction of prokaryotic expression vectors

| **Primer Name** | **Primer sequence（5ʹ-3ʹ）** | **Length (bp)** | |
| --- | --- | --- | --- |
| pCold^TM^TF-*AtATO*-F | ATGGAGCTCGGTACCATGTCGTCGACTCTCCTCGAG | | 1515 |
| pCold^TM^TF-*AtATO*-R | GACAAGCTTGAATTCTCAGATGAGACCTTGGCGTTG | |  |
| pCold^TM^TF-*AtCC1*-F | ATGGAGCTCGGTACCATGGTAGAGAAAGTTTCTAGTATC | | 1263 |
| pCold^TM^TF-*AtCC1*-R | GACAAGCTTGAATTCTTAAACGACCTTACTCTCGG | |  |
| pCold^TM^TF-*AtCC1-LIKE*-F | ATGGAGCTCGGTACCATGGAGTTTGACGAATACGAGTACCTG | | 1584 |
| pCold^TM^TF-*AtCC1-LIKE*-R | GACAAGCTTGAATTCTCAGGCCTTGGCTTTGGCTTCATATTC | |  |
| pCold^TM^TF-*AtRSZ22A*-F | ACCCTCGAGGGATCCATGTCGCGTGTGTATGTTGG | | 591 |
| pCold^TM^TF-*AtRSZ2*2A-R | AGACTGCAGGTCGACTCAGCTCCGGCTTCTGCGCAC | |  |
| pCold^TM^TF-*AtCBP80*-F | ATGGAGCTCGGTACCATGAGCAATTGGAAAACTC | | 2547 |
| pCold^TM^TF-*AtCBP80*-R | AGACTGCAGGTCGACTTATGTAATGGGAATTGAAG | |  |
| pCold^TM^TF-*AtSWAP*-F | ATGGAGCTCGGTACCATGTTCAGCTCGATGCAGATACTG | | 2358 |
| pCold^TM^TF-*AtSWAP*-R | GACAAGCTTGAATTCCTATCTCTTTCTCCCACCACGTTCC | |  |
| pCold^TM^TF-*AtMOS4*-F | ATGGAGCTCGGTACCATGGCGACGAACAATGGTGATGT | | 762 |
| pCold^TM^TF-*AtMOS4*-R | GACAAGCTTGAATTCTCATTGCATTTGAAGTGGCTCG | |  |
| XF245-*AtCC1*-F | ATGGAGGCCGGATCCATGGATGAGAAAGTTTCTAGTATC | | 1263 |
| XF245-*AtCC1*-R | CTGCAGGACGAATTCTTAAACGACCTTACTCTCGG | |  |
| XF245-*AtCC1-LIKE*-F | ATGGAGGCCGGATCCATGGAGTTTGACGAATACGAGTACCTG | | 1584 |
| XF245-*AtCC1-LIKE*-R | CTGCAGGACGAATTCTCAGGCCTTGGCTTTGGCTTCATATTC | |  |
| pCold^TM^TF-*BraATO2*-F | AGGCATATGGAGCTCATGTCGTCGACTCTCCTCGA | | 1521 |
| pCold^TM^TF-*BraATO2*-R | CTTGAATTCGGATCCTCAGATGAGACCTTGACGTTGG | |  |
| *BraATO2^C413A^-F* | GAAATTTGAAGCAGAGATATGTG | | 282  1239 |
| *BraATO2^C413A^-R* | CACATATCTCTGCTTCAAATTTC | |  |
| *BraATO2^C416A^-F* | ATGCGAGATAGCCGGAAACAGTG | | 273  1248 |
| *BraATO2^C416A^-R* | CACTGTTTCCGGCTATCTCGCAT | |  |
| pET-28a (+) -sumo-*BraATO2-*F | ATGGGTCGCGGATCCATGTCGTCGACTCTCCTCGA | | 1521 |
| pET-28a (+) -sumo-*BraATO2-R* | TTGTCGACGGAGCTCTCAGATGAGACCTTGACGTTGG | |  |

**Supplementary Table 2.** Primers for the acquisition and identification of *B. rapa* (Chinese cabbage) *BraATO2* overexpression and VIGS-mediated silenced lines.

| **Primer Name** | **Primer sequence（5ʹ-3ʹ）** | **Length (bp)** |
| --- | --- | --- |
| XF350-*BraATO2*-F  XF350-*BraATO2*-R | GCCGGATCCGATTATATGTCGTCGACTCTCCTCGA  GTGGCTAGCGAATTCTCAGATGAGACCTTGACGTTGG | 1521 |
| RT-*BraATO2*-F  RT-*BraATO2*-R | TGATGCCAGTGCGGACTATG  CCTTGAAAACTTCAGTTTACGCGAT | 208 |
| RT-*BraACTIN*-F  RT-*BraACTIN*-R | TCAGGTGTCCCGAGGTTCT  TACTCATCCTATCAGCAATCCC | 180 |
| pTYs-CP-F  pTYs-CP-R | TCCACCCTCACCACCTTC  GGGACAGACCTCGCTAACT | 552 |
| RT-*AtACTIN*-F  RT-*AtACTIN*-R | CTCAGCACCTTCCAACAGATGTGGA  CCAAAAAAATGAACCAAGGACCAAA | 364 |

**Supplementary Table 3.** Summary statistics for clean data obtained from transcriptome sequencing of *B. rapa* (Chinese cabbage )

| **SampleID** | **ReadNum** | **BaseNum** | **N50** | **MeanLength** | **MaxLength** | **MeanQscore** |
| --- | --- | --- | --- | --- | --- | --- |
| A1 | 1,618,609 | 1,952,403,336 | 1,329 | 1,206 | 21,999 | Q12 |
| A2 | 2,488,541 | 3,093,072,821 | 1,391 | 1,242 | 21,257 | Q12 |
| A3 | 1,904,05 | 2,339,506,79 | 1,379 | 1,228 | 115,898 | Q12 |
| S1 | 1,725,358 | 2,141,588,142 | 1,390 | 1,241 | 17,599 | Q12 |
| S2 | 1,893,915 | 2,324,740,240 | 1,382 | 1,227 | 376,692 | Q12 |
| S3 | 1,923,264 | 2,400,971,999 | 1,404 | 1,248 | 17,884 | Q12 |
| W1 | 2,683,130 | 3,237,440,221 | 1,324 | 1,206 | 14,572 | Q12 |
| W2 | 2,426,537 | 3,007,259,118 | 1,385 | 1,239 | 51,566 | Q12 |
| W3 | 2,104,828 | 2,562,919,219 | 1,367 | 1,217 | 54,371 | Q12 |

Note: **SampleID:** Sample Name (An illustration of three treatments applied to Chinese cabbage, including A (HA, an inhibitor of H_2_S synthesis), S (H_2_S), W (wild-type). Each treatment was biologically replicated three times. **ReadNum:** Total count of sequences; **BaseNum:** Total count of bases; **N50:** Length of the N50 ; **MeanLength:** Average length of read; **MaxLength:** Maximum length of reads; **MeanQscore:** Average quality score of sequences.

**Supplementary Table 4.** Statistical table of full-length sequence data

| **SampleID** | **Number of clean reads**  **(except rRNA)** | **Number of full**  **length reads** | **Full-Length**  **Percentage (FL%)** |
| --- | --- | --- | --- |
| A1 | 1,418,695 | 1,258,553 | 88.71% |
| A2 | 2,229,803 | 1,986,821 | 89.10% |
| A3 | 1,695,056 | 1,503,696 | 88.71% |
| S1 | 1,512,077 | 1,333,436 | 88.19% |
| S2 | 1,688,877 | 1,489,853 | 88.22% |
| S3 | 1,730,551 | 1,537,748 | 88.86% |
| W1 | 2,383,775 | 2,111,678 | 88.59% |
| W2 | 2,162,777 | 1,911,332 | 88.37% |
| W3 | 1,869,007 | 1,651,761 | 88.38% |

Note: **SampleID** represents the identifier for each sample (A: HA; S:H_2_S; W:WT). **Number of clean reads (except rRNA):** the count of sequences remaining after ribosomal RNA removal. **Number of full-length reads:** the total count of reads that cover the entire length of the sequence. **Full-Length Percentage (FL%):** the proportion of the total sequencelength that consists of full-length reads.

**Supplementary Table 5.** Comprehensive analysis of alternative splicing events in the full-length transcriptome of *B. rapa* (Chinese cabbage).

(Details are provided in an Excel format file)

**Supplementary Table 6.** Primers for splicing pattern analysis

| **Primer Name** | **Primer sequence（5ʹ-3ʹ）** | **Length (bp)** |
| --- | --- | --- |
| *BraAGL31* (*Bra031888*)-F | ATGGGGAGAAGAAAAGTAGAGATC | 693/750 |
| *BraAGL31* (*Bra031888*)-R | TTAGAGCAAGATAAAAATGGTTTTGAGA |  |
| *BraGRP8* (*Bra011869*)-F | ATGTCTGCAGAAGTAGAGTACC | 136/278 |
| *BraGRP8* (*Bra011869*)-R | TCCCAGTCTCACGATCGTTA |  |
| *BraARF1* (*Bra012376*)-F | GACCGTGTTGTTGAGGCCAG | 172 |
| *BraARF1* (*Bra012376*)-R | CTGAATGTACCAGTGACGCTG |  |
| *BraFER1* (*Bra005677*)-F | TTCAAGGAATCAAGTGATGAAG | 117 |
| *BraFER1* (*Bra005677*)-R | TTCAAAATCAGAGATAGGGGAGAC |  |
| *BraFER3* (*Bra003226*)-F | CTTTCCCTCGCTCGACATTT | 156 |
| *BraFER3* (*Bra003226*)-R | CTTGAAAAACTTGGCAAGACC |  |
| *BraLOG1* (*Bra034399*)-F | GCCATGTCATCGGGGTTATCC | 91 |
| *BraLOG1* (*Bra034399*)-R | TGCATGTCTGCTACTGCCTTC |  |
| *AtAGL31* (*AT5G65050*)*-F* | GTCCAAAGGTTAGCACAAAGACA | 136 |
| *AtaAGL31* (*AT5G65050*)*-R* | GTATCCACACTTGCATTATCGACA |  |

| **Supplementary Table 7.** *B. rap*a (Chinese cabbage) genetic transformation medium formula |
| --- |
| \| **Medium type** \| **Culture medium formulation** \| \| --- \| --- \| \| Seed culture medium \| 1/2 MS (3% sucrose, 0.8% agar) \| \| Preculture medium \| 1/2 MS + 6-BA 2 mg/L + NAA 0.1 mg/L \| \| Co-cultivation medium \| 1/2 MS + 6-BA 2 mg/L + NAA 0.1 mg/L + AS 10 mg/L \| \| Differential medium \| 1/2 MS + 6-BA 2 mg/L + NAA 0.1 mg/L + TMT 200 mg/L + Kan^+^ 10 mg/L \| \| Rooting medium \| 1/2 MS + NAA 0.1 mg/L + TMT 200 mg/L + Kan^+^ 10 mg/L \| |
